# Microanalysis, Pharmacokinetics and Tissue Distribution of Polysaccharide-Protein Complexes from Longan Pulp in Mice

**DOI:** 10.3390/ijms161024403

**Published:** 2015-10-15

**Authors:** Ting Min, Jie Sun, Yang Yi, Hong-Xun Wang, Fei Hang, You-Wei Ai, Li-Mei Wang

**Affiliations:** 1 College of Food Science & Engineering, Wuhan Polytechnic University, Wuhan 430023, China; E-Mails: minting1323@163.com (T.M.); qiqijiayuguan@163.com (J.S.); wanghongxunhust@163.com (H.-X.W.); aywlingyun@126.com (Y.-W.A.); wanglimeiyx@163.com (L.-M.W.); 2 Hubei Collaborative Innovation Center for Processing of Agricultural Products, Wuhan 430023, China; 3 Sericultural & Agri-food Research Institute, Guangdong Academy of Agricultural Sciences, Key Laboratory of Functional Foods, Ministry of Agriculture, Guangzhou 510610, China; E-Mail: hf1311@163.com

**Keywords:** longan pulp, polysaccharide-protein complex, HPSEC-FD, pharmacokinetics, distribution

## Abstract

A high performance size exclusion-fluorescence detection (HPSEC-FD) method combined with fluorescein isothiocyanate (FITC) prelabeling was established for the microanalysis of polysaccharide–protein complexes from longan pulp (LPP). FITC-labeled LPP (LPPF) was fractionated by gel filtration chromatography. The weight-average molecular weight and FITC substitution degree of LPPF were 39.01 kDa and 0.20%, respectively. The HPSEC-FD calibration curves linear over the range of 1–200 µg/mL in mouse plasma, spleen and lung samples with correlation coefficients greater than 0.995. The inter-day and intra-day precisions of the method were not more than 6.9%, and the relative recovery ranged from 93.7% to 106.4%. The concentration–time curve of LPPF in plasma following intravenous (i.v.) administration at 40 mg/kg body weight well fitted to a two-compartment model. LPPF rapidly eliminated from plasma according to the short half-lives (*t*_1/2α_ = 2.23 min, *t*_1/2β_ = 39.11 min) and mean retention times (*MRT*_0–*t*_ = 1.15 h, *MRT*_0–∞_ = 1.39 h). After administration over 5 to 360 min, the concentration of LPPF in spleen homogenate decreased from 7.41 to 3.68 µg/mL; the concentration in lung homogenate decreased from 9.08 to 3.40 µg/mL. On the other hand, the increasing concentration of LPPF fraction with low molecular weight in heart homogenate was observed.

## 1. Introduction

Longan fruit (*Dimocarpus* *longan* Lour*.*) has been traditionally used in Chinese medicinal formulation for a long time, serving as a common agent to promote blood metabolism, soothe nerves, relieve insomnia and prevent amnesia [1,2]. The extraction, purification, structure and bioactivity of the polysaccharides and polysaccharide-protein complexes (major bioactive ingredients) of longan pulp have been well studied [2,3,4,5,6,7,8]. Longan polysaccharide-protein complexes (LPP) can significantly enhance the immune functions of mice, such as antibody production against chicken red blood cells, ConA-induced splenocyte proliferation, macrophage phagocytosis, natural killer (NK) cell cytotoxicity against YAC-1 lymphoma cells and cytokine secretion in serum [4,8]. However, the potential mechanism related to their pharmacokinetics and tissue distributions in experimental animals is still unclear. In most cases, polysaccharides are studied as the carrier moiety of micromolecular drug (or natural bioactive ingredient), however their *in vivo* mechanism have rarely been investigated due to a lack of specific microassay methods [9,10].

Most of the published studies on the pharmacokinetics and tissue distribution of polysaccharides used analytical methods including fluorescence labeling combined with chromatography [9,11,12], isotope labeling [13,14], spectrophotometry [15,16], fluorospectrophotometry [17,18] and biological assay [19]. In view of the intrinsic characteristics of analyte and the sensitivity and specificity of the analytical method, the combination of high performance size exclusion chromatography (HPSEC) with fluorescent prelabeling was thought to be the most feasible method for the quantitative determination of LPP, especially in biological samples. Fluorescein isothiocyanate (FITC), which could combine with amino groups through the isothiocyano residue under weak alkali conditions [12], might be an effective labeling agent for the binding protein of LPP. Accordingly, a HPSEC-fluorescence detection (HPSEC-FD) method for the quantitative determination of LPP was established based on FITC prelabeling and followed with an investigation of the pharmacokinetics and tissue distribution in mice.

## 2. Results and Discussion

### 2.1. Purification of Longan Polysaccharide Complexes (LPP) and Fluorescein Isothiocyanate-Labeled LPP (LPPF)

**Figure 1:**
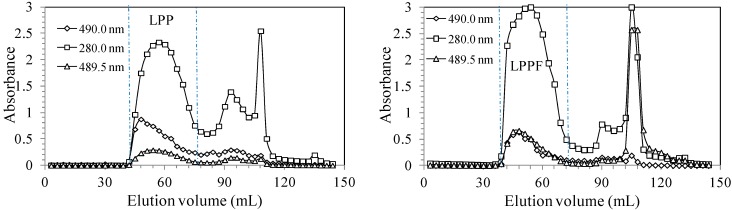
Gel filtration chromatograms of crude longan polysaccharide-protein complexes treated with (**Right**) or without (**Left**) Fluorescein isothiocyanate (FITC).

**Figure 2:**
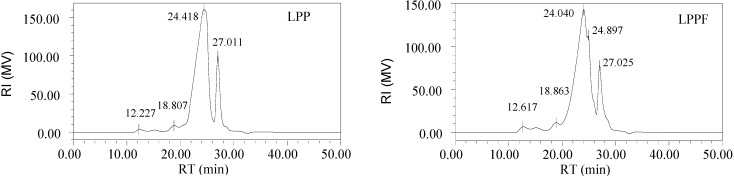
High performance size exclusion chromatograms of longan polysaccharide-protein complexes (LPP) (**Left**) and Fluorescein isothiocyanate-labeled LPP (LPPF) (**Right**) obtained by refractive index detection.

Crude polysaccharide-protein complexes from longan pulp pretreated with or without FITC were fractionated on a Sephadex G-100 gel column to obtain the main fraction named LPPF or LPP (Figure 1). The signals detected at 490 nm (phenol-sulfuric acid assay) and at 280 nm (spectrophotometer) belonged to polysaccharides and proteins, respectively. LPP were identified by the simultaneous absorbance peaks at 490 and 280 nm [20,21]. FITC showed the characteristic peaks at 280.0 and 489.5 nm (data not shown). As seen in Figure 1, the absorbances of LPP at 280 and 489.5 nm both increased after treatment with FITC indicating that LPPF were the FITC labeled derivatives of LPP [22]. The weight-average molecular weights (*M*_W_) of LPP and LPPF were estimated to be 37.52 and 39.01 kDa, respectively. Their molecular weight distributions exhibited no obvious difference (Figure 2).

### 2.2. Spectral Features of LPP and LPPF

**Figure 3:**
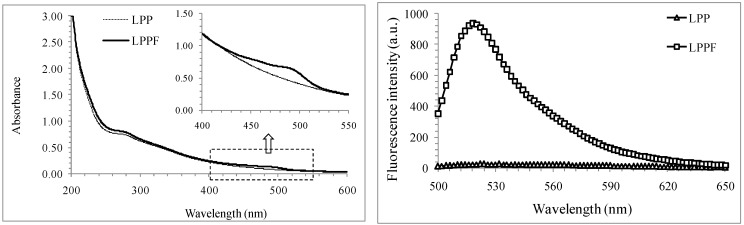
Ultraviolet-visible (**Left**) and fluorescence spectra (**Right**) of LPP and LPPF. The excitation wavelength for fluorescence scanning was 495 nm.

As seen in the Ultraviolet-visible (UV-vis) spectra of LPP and LPPF (Figure 3), the absorbances of LPPF at about 490 and 280 nm were obviously stronger than those of LPP. It was consistent with the comparative analysis on their gel filtration chromatograms. In addition, only LPPF showed an evident fluorescence peak with peak value at 520 nm. The emission wavelength and excitation wavelength for the HPSEC-FD method of LPPF were, respectively, 520 and 495 nm, which were same as the FITC-labeled arabinogalactan and chitosan [9,23].

The linear regression equation of FITC concentration (*c*, µg/mL) *vs.* absorbance (*A*) was established as follow: *A* = 0.202*c* − 0.003 (*R*^2^ = 0.999). The FITC substitution degree (%) of LPPF was calculated to be 0.20%, which was significantly lower than that of FITC-labeled chitosan [23].

### 2.3. Method Validation

**Figure 4:**
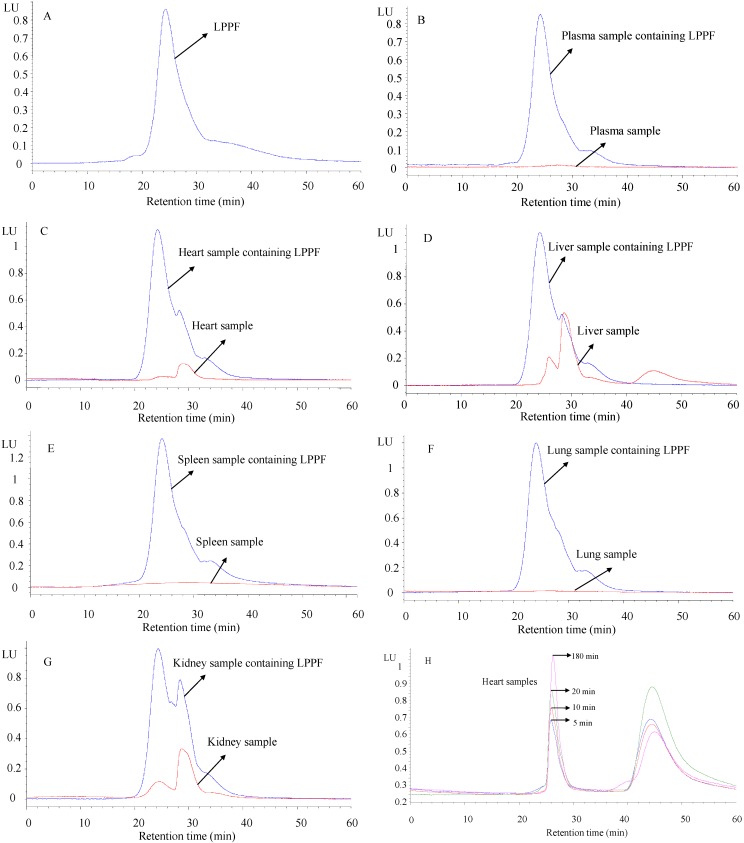
High performance size exclusion chromatograms of LPPF obtained by FD. (**A**) The chromatogram of 50 µg/mL LPPF prepared by phosphate buffer (pH 7.4); (**B**–**G**) The chromatograms of biological samples mixed with or without 50 µg/mL LPPF; (**H**) The chromatograms of heart samples from mice at predefined times (5, 10, 20 and 180 min) after a single intravenous (i.v.) administration of LPPF (40 mg/kg body weight).

Method specificity was demonstrated by assessing the interferences of endogenous substances from prepared biological samples. The chromatograms of LPPF obtained by FD in the presence and in the absence of interferences are shown in Figure 4. The typical peak value of LPPF was approximately 24.8 min (Figure 4A), which was consistent with the chromatogram obtained by refractive index detection (RID). The processed samples of mouse plasma, spleen and lung all exhibited no responsive signal in the chromatograms, *i.e.*, they had no endogenous substance that significantly impacted on the detection of LPPF (Figure 4B,E,F). The characteristic peaks of other processed biological samples at about 28.1 min were partly overlapped with that of LPPF (Figure 4C,D,G). It was indicated that the proposed method could be only used for the microanalysis of LPPF in mouse plasma, spleen and lung. Likewise, the study of Lin *et al.* [11] also confirmed that blank plasma had no signal responding to fluorescence detection, but blank heart tissue sample had. FITC-labeled *Ophiopogon japonicas* polysaccharides [24] could be well-separated from the interferents of heart due to their lower molecular weights (4.8 kDa) compared with LPPF. The biodisposition of FITC-labeled arabinogalactan in rats was investigated by detecting the fluorescence intensities of tissue samples [9], as well as that of FITC-labeled chitosan in mice [23]. However, the quantitative analyses of these polysaccharides might be interfered by the fluorescent substances derived from murine tissues including heart, liver and kidney. It was also suggested that the concentrations of FITC-labeled polysaccharides in mouse plasma, spleen and lung samples could be indirectly determined by the fluorescence intensity.

The standard curve of peak area (*y*, arbitrary unit) to LPPF concentration (*x*, µg/mL) was constructed using the linear least squares regression model. The standard curves, correlation coefficients and linear ranges of quantitative determination of LPPF in the samples of plasma, spleen and lung are listed in Table 1. The HPSEC-FD method of LPPF showed good linear responses in the concentration range of 1–200 µg/mL, as the correlation coefficients (*R*^2^) were larger than 0.995. The limit of detection (DL) and the limit of quantization (QL) were 0.25 and 0.85 µg/mL, respectively. The values were lower than those of phenol-sulfuric acid method (DL 1.54 μg/mL, QL 4.67 µg/mL) [25], but were higher than those of the HPSEC-FD method of FITC-labeled *Radix Ophiopogonis* polysaccharide due to the relatively lower FITC substitution degree of LPPF [11].

**Table 1:** Calibration curves for Fluorescein isothiocyanate-labeled longan polysaccharide-protein complexes (LPPF) in mouse plasma and tissue homogenate supernatants.

Biological Samples	Standard Curves	Correlation Coefficients	Linear Ranges * (µg/mL)
Blank	*y* = 2.199*x* + 2.273	0.998	1–200 µg/mL
Plasma	*y* = 2.223*x* + 1.034	0.996	1–200 µg/mL
Spleen	*y* = 2.349*x* − 0.485	0.996	1–200 µg/mL
Lung	*y* = 2.138*x* + 2.616	0.995	1–200 µg/mL

* Data were obtained at six concentrations, and each repeated in triplicate.

Precision of the proposed method was explored by evaluating the peak areas of different concentrations of LPPF, to measure the agreement between test results from multiple and repeated instrumental analysis. As shown in Table 2, the intra-day precision and inter-day precision were basically in accordance with testing requirement (*RSD* < 5%). Accuracy evaluated by recovery test can measure the amount of analyte that is quantified relative to the amount present in the sample. The relative recovery values ranging from 93.7% to 106.4% were acceptable (Table 2) [11].

**Table 2:** Precision and accuracy for quantitative determination of LPPF by high performance size exclusion-fluorescence detection (HPSEC-FD) method.

Biological Samples	Added Concentration (µg/mL)	Relative Recovery (%)	Precision (*RSD*, %)	
			Intra-Day	Inter-Day
Blank	2	99.4 ± 4.3	3.4	4.0
	40	101.7 ± 3.0	2.4	2.9
	80	101.4 ± 2.3	3.1	4.2
Plasma	2	98.2 ± 5.1	4.5	4.5
	40	102.3 ± 4.1	3.2	2.9
	80	103.4 ± 5.2	5.1	2.6
Spleen	2	106.4 ± 3.9	3.6	3.5
	40	96.7 ± 5.7	4.4	5.0
	80	105.4 ± 3.3	5.7	3.5
Lung	2	93.7 ± 2.9	3.0	4.2
	40	104.2 ± 3.1	4.4	3.8
	80	96.5 ± 3.4	2.5	6.9

### 2.4. Pharmacokinetics and Tissue Distribution of LPPF

**Figure 5:**
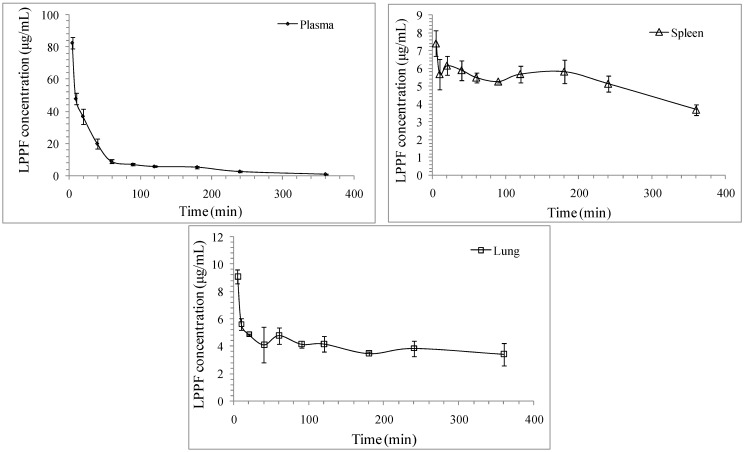
LPPF concentration *vs.* time profile following intravenous (i.v.) administration at 40 mg/kg body weight. Data are represented as mean ± standard derivation (*n* = 5).

The concentration–time curve of LPPF in mice plasma following i.v. administration at 40 mg/kg body weight well fitted to a two-compartment model (*R*^2^ = 0.99 ± 0.01, *n* = 5). LPPF injected intravenously might be first distributed from central compartment into peripheral compartments to a pseudo-equilibrium (alpha phase), followed by a process of gradual elimination from plasma primarily attributed to excretion (beta phase), resulting in the biphasic decrease of plasma concentration of LPPF as shown in Figure 5. The important pharmacokinetics parameters of LPPF are summarized in Table 3. The concentration-time curves of bioactive polysaccharides in animal plasma were mostly confirmed to be two-compartment model, but their pharmacokinetics parameters showed significant differences due to different physicochemical characteristics, administration methods and dosages [10]. A typical two-compartment model of lentinan (50 kDa) in mice plasma after intravenous administration was also identified [26]. In addition, the half-lives of distribution phase and elimination phase (*t*_1/2α_ and *t*_1/2β_) of lentinan in beagle dogs after i.v. administration showed positive correlations with dosage [27]. It was suggested that the reason for the longer *t*_1/2α_ (1.27 h) and *t*_1/2β_ (2.64 h) of lentinan in mice plasma might be related to the much lower administration dosage (0.5 mg/kg body weight), compared with LPPF. The results from clinical investigations indicated that most of the polysaccharides used for anti-cancer drug precursor had the *M*_W_ ranging from 25 to 50 kDa, and the reasons for such a situation were closely related to the anti-cancer activity and pharmacokinetic behavior of polysaccharide [28]. Polysaccharides with larger *M*_W_ usually exhibited lower clearances and longer mean retention times (MRT) [10,29,30,31]. The correlation of molecular size to clearance and mean retention time may partially explain why lentinan had longer mean retention times (*MRT*_0–t_ = 2.78 h, *MRT*_0–∞_ = 3.01 h) compared with LPPF [26], while *Radix Ophiopogonis* polysaccharide (4.8 kDa) showed a shorter one (39.0 min) [11].

LPPF exhibited short half-lives in plasma, implying that LPPF could be rapidly transferred to peripheral tissues or excreted in urine. As shown in Figure 5, the LPPF concentration of spleen homogenate supernatant was measured to be 7.41 µg/mL at 5 min after administration, and then significantly decreased (*p* < 0.05). The concentrations ranging from 10 to 240 min exhibited no obvious difference (*p* > 0.05), but were all significantly higher than that at 360 min (*p* < 0.05). The LPPF concentration–time curve of lung homogenate supernatant was similar to that of spleen homogenate supernatant, as the highest value was 9.08 µg/mL at 5 min after administration. The results were close to those of *Radix Ophiopogonis* polysaccharide [11], and LPPF might be mainly distributed to kidney. The LPPF concentration in kidney could not be measured in the present study. However, deep yellow urine, which might contain a high concentration of LPPF, was repeatedly observed on mice sacrificed at 5 and 10 min after administration. The pharmacokinetics and biodisposition of polysaccharides were closely related to their molecular weights and net charges [10]. LPPF and LPP showed no significant difference in molecular weight. The charge only impacted on the uptake of polysaccharide by hepatocytes and tumor cells [10]. It could be concluded that the effect of FITC on the pharmacokinetics and biodisposition of LPP was negligible.

As seen in Figure 4H, the content of LPPF in heart might gradually increase after administration, according to the chromatograms of heart samples obtained at predefined times (5, 10, 20, 180 min). This situation was different with the reports from Lin [11] and Zhang [26]. The factions of LPPF accumulated in heart showed smaller *M*_W_, as the retention time of peak value (25.90 min) was in accordance with the *M*_W_ of 1.41 kDa. According to the appearance of new peak at about retention time 45 min, the binding FITC of LPPF was speculated to be partly dissociated in heart. Chitosans with different *M*_W_ (112.3 and 496.2 kDa) exhibited different tissue distributions in rats [18]*.* A few studies confirmed that the liver accumulation and kidney excretion of polysaccharides were related to *M*_W_ [16,32,33]; polysaccharide selectivity of heart responding to *M*_W_ was rarely reported.

**Table 3:** Pharmacokinetics parameters of LPPF after a single intravenous (i.v.) administration at 40 mg/kg (mean ± SD, *n* = 5).

Parameters *	Values
Half-life of distribution phase *t*_1/2α_ (min)	2.23 ± 0.35
Half-life of elimination phase *t*_1/2β_ (min)	39.11 ± 2.73
Apparent volume of distribution of the central compartment *V*_1_ (L/kg)	0.09 ± 0.01
Apparent volume of distribution of the peripheral compartment *V*_2_ (L/kg)	0.26 ± 0.03
Clearance of the central compartment *CL*_1_ (L/h/kg)	0.53 ± 0.03
Clearance of the peripheral compartment *CL*_2_ (L/h/kg)	0.60 ± 0.05
Area under concentration–time curve *AUC*_0–t_ (mg·h/L)	56.80 ± 3.44
Area under concentration–time curve *AUC*_0–∞_ (mg·h/L)	58.85 ± 3.51
Mean retention time *MRT*_0–t_ (h)	1.15 ± 0.04
Mean retention time *MRT*_0–∞_ (h)	1.39 ± 0.08

* The parameters including *t*_1/2α_, *t*_1/2β_, *V*_1_, *V*_2_, *CL*_1_ and *CL*_2_ were calculated by a two-compartmental moment analysis, and the other parameters were calculated by non-compartmental moment analysis.

## 3. Experimental Section

### 3.1. Materials

Fresh longan fruits (cv*.* Chu-Liang) were provided by Sericulture & Agri-Food Research Institute of Guangdong Academy of Agricultural Sciences (Guangzhou, China). Longan pulps were manually stripped and hot-air dried at 75 °C for 50 h. The dried pulps were stored at −20 °C until use.

### 3.2. Animals and Biological Sample Preparation

Eight weeks old Kunming mice (specific pathogen-free, male) were purchased from Lab Animal Center of Huazhong University of Science and Technology (Wuhan, China), and were kept in an environmentally controlled breeding room for a week before experiments. They were freely fed with standard laboratory food and water. All relative protocols and procedures were approved by our Institutional Animal Care and Use Committee, and were performed in accordance to the Guide for the Care and Use of Laboratory Animals.

Mice were sacrificed by bloodletting via eyeball to collect blood into a 1.5 mL heparin-soaked Eppendorf tube. After centrifugation at 3000 rpm/min for 10 min, the separated plasma was frozen at −20 °C until analysis. Various tissues including heart, liver, spleen, lung and kidney were immediately harvested. Each organ was homogenized with a glass homogenizer (6 cm diameter) using a 3-fold volume of 0.1 mol/L phosphate buffer (pH 7.4) [11]. The homogenate was then centrifuged at 10,000 rpm/min for 5 min to collect supernatant. The supernatant was stored at −20 °C until analysis.

### 3.3. Polysaccharide–Protein Complex Preparation

Crude polysaccharide-protein complexes of longan pulp were isolated according to our previous method [6]. One hundred and fifty milligram complexes were dissolved in 10 mL sodium carbonate buffer solution (0.5 mol/L, pH 8.3) with or without 10 mg FITC [22]. After keeping the mixture in dark at room temperature for 24 h, supernatants were separated by centrifugation at 4500 rpm/min for 10 min. Three milliliter supernatants were injected onto a Sephadex G-100 gel column (1.6 cm × 50 cm, gel was purchased from Pharmacia Biotech (Piscataway, NJ, USA)), followed by the elution with distilled water at a flow rate of 0.3 mL/min. Three milliliter per tube of eluants were collected for determining the concentrations of polysaccharide, protein and FITC by phenol-sulfuric acid method (490 nm) [34] and spectrometry (280 and 489.5 nm), respectively. The eluants containing the main fraction, which was identified by gel filtration chromatogram, were vacuum-concentrated at 55 °C using a rotary evaporator (BC-R203, Shanghai Biochemical Equipment Co., Shanghai, China) and then freeze-dried to obtain FITC-labeled or unlabeled longan polysaccharide-protein complexes (*i.e.*, LPPF and LPP). The yield of LPP from the crude complexes was 74.78%. The polysaccharide content and protein content of LPP were 96.54% and 2.94%, respectively.

### 3.4. Spectrum Analysis

Polysaccharide-protein complexes were dissolved in distilled water to the concentration of 0.5 mg/mL. After centrifugation at 4500 rpm/min for 15 min, the supernatant was scanned in the wavelength range of 200–600 nm using a UV-vis spectrophotometer (UV 1800, Shimadzu Corporation, Kyoto, Japan) and in the emission wavelength range of 500–700 nm (excitation wavelength 490 nm) using a fluorescence spectrophotometer (FP-6500, Jasco Corporation, Kyoto, Japan).

A series of FITC solutions with concentrations in the range of 0.1–5.0 µg/mL was prepared by distilled water. Distilled water was used for zero setting. The absorbance of FITC solution was measured at 489.5 nm by a UV-vis spectrophotometer. The standard curve and regression equation of FITC concentration *vs.* absorbance were established. The absorbance difference between 50 µg/mL LPPF and 50 µg/mL LPP was used in the regression equation to calculate the concentration of binding FITC. The FITC substitution degree (%) of LPPF was expressed as the mass percentage of binding FITC to LPPF.

### 3.5. HPSEC Analysis

#### 3.5.1. Detection Conditions

HPSEC analysis was performed with a Waters Series system containing a refractive index detector (2414), a fluorescence detector (2475), a binary pump (1525) and an Ultrahydrogel 1000 SEC column (7.8 mm × 300 mm, Waters, Milford, MA, USA). The excitation wavelength and emission wavelength of FD were 495 and 520 nm, respectively. Moreover, mobile phase was 0.1 mol/L sodium nitrate, flow rate was 0.4 mL/min, injection volume was 25 µL, and column temperature was 35 °C.

Polysaccharide samples were dissolved in 0.1 mol/L sodium nitrate to the concentration of 2 mg/mL. The solutions were injected onto the SEC column after filtrating through a 0.45 µm filter membrane and then detected using the refractive index detector. To estimate the *M*_W_ of LPP and LPPF, poly(ethylene oxide) standards with known *M*_W_ (2.42 × 10^4^, 4.13 × 10^4^, 6.75 × 10^4^, 1.49 × 10^5^ and 3.13 × 10^5^ kDa, Waters, Milford, MA, USA) were used for calibration.

#### 3.5.2. Selectivity

LPPF samples were dissolved in phosphate buffer (pH 7.4) to the concentration of 500 µg/mL. Twenty microliter LPPF solutions were added to 180 µL blank plasma or 180 µL tissue homogenate supernatant. The mixture was then treated according to the modified method of Kaneo *et al.* [9]. In brief, 200 µL of the mixture were mixed with 80 µL of 30% (*w*/*v*) trichloroacetic acid. After centrifugation at 14,000 rpm/min for 5 min, the supernatant (200 µL) was neutralized by addition of 30 µL 11% (*w*/*v*) of NaOH. After filtration, the biological sample containing or not containing 50 µg/mL LPPF was analyzed by the HPSEC-FD method.

#### 3.5.3. Quantitative Determination

A series of standard LPPF solutions with concentrations in the range of 10–2000 µg/mL were prepared by phosphate buffer (pH 7.4). Twenty microliter LPPF solutions were mixed with 180 µL blank plasma or 180 µL tissue homogenate supernatant and treated with the method as described in Section 3.5.2 to obtain the standard calibration samples with six levels ranging from 1 to 100 µg/mL. The peak areas and retention times were used to establish a linear standard curve and the linearity correlation coefficient (*R*^2^) was calculated by regression analysis. DL and QL were calculated as the lowest level of LPPF that resulted in a signal-to-noise ratio of 3 and 10, respectively.

#### 3.5.4. Precision and Accuracy

To evaluate the precision and accuracy of the method, repeatability (intra-day measurements) and intermediate precision (inter-day measurements) were examined by adding known levels of LPPF (4, 40 and 80 µg/mL). Repeatability was calculated from five replicate determinations of sample solution containing 20 µg/mL LPPF on same day. Intermediate precision was analyzed by the same experiment additionally repeated on three consecutive days. Precision and accuracy were calculated using the formulas described by Skidana *et al.* [35].

### 3.6. Animal Experiment

The mice, weighing 22–26 g, were fasted for 12 h prior to experiments. LPPF samples were dissolved in saline to the final concentration of 5 mg/mL for injection after filtration through a 0.45 µm filter membrane. Mice were administrated with a single dose (40 mg/kg body weight) of LPPF solution through caudal vein, and then were sacrificed by bloodletting via eyeball at predefined times (5, 10, 20, 40, 60, 90, 120, 180, 240 and 360 min; *n* = five per time point) to collect bloods, spleens and lungs. Tissue homogenates were prepared by the procedures described by Lin *et al.* [11]. Twenty microliter phosphate buffer solutions were mixed with 180 µL plasma or 180 µL tissue homogenate supernatant. The mixture was then pretreated with the modified method of Kaneo *et al.* (as described in Section 3.5.2) [9] for HPSEC determination.

### 3.7. Data Analysis

Data were expressed as means ± standard derivation (SD). Significance of difference (*p* < 0.05) was evaluated with one-way ANOVA followed by the Student–Newman–Keuls test using IBM SPSS Statistics 19 software (IBM, Armonk, NY, USA). The DAS 2.0 pharmacrokinetic program (Chinese Pharmacology Society, Shanghai, China) was used to calculate pharmacokinetic parameters.

## 4. Conclusions

The combination of FITC prelabeling and HPSEC-FD makes the quantitative determination of LPP possible in mouse plasma, spleen and lung samples. Based on the HPSEC-FD method, a typical two-compartment model of LPPF in mice plasma after intravenous administration was identified, and a small amount of LPPF were confirmed to be distributed to spleen and lung. Because of the impact of endogenous interferences from other tissues on the microanalysis method, the distribution of LPPF in mice has not been clearly explored. The proposed HPSEC-FD method needs to be modified for better sensitivity and specificity. Moreover, the effects of structure on the pharmacokinetics and tissue distribution of polysaccharide-protein complexes from longan pulp should be further investigated.
